# 电视辅助胸腔镜手术和体部立体定向放疗治疗早期非小细胞肺癌的临床疗效比较

**DOI:** 10.3779/j.issn.1009-3419.2016.03.04

**Published:** 2016-03-20

**Authors:** 建东 王, 占杰 左, 洪波 张, 伟 李, 坤峰 王

**Affiliations:** 100027 北京，武警北京总队医院胸部肿瘤治疗中心 Thoracic Cancer Treatment Center, Armed police Beijing Corps Hospital, Beijing 100027, China

**Keywords:** 肺肿瘤, 电视辅助胸腔镜手术, 体部立体定向放疗, 生存率, *Meta*分析, Lung neoplasms, Video-assisted thoracoscopic surgery, Stereotactic body radiotherapy, Survival rate, *Meta*-analysis

## Abstract

**背景与目的:**

越来越多的胸科医生选择电视辅助胸腔镜手术（video-assisted thoracoscopic surgery, VATS）来治疗早期非小细胞肺癌（non-small cell lung cancer, NSCLC）。目前，仍然没有随机试验来比较VATS和体部立体定向放疗（stereotactic body radiotherapy, SBRT）的临床疗效。为此，在本*meta*分析中，我们比较了电视辅助胸腔镜手术和体部立体定向放疗在治疗早期NSCLC患者过程中的疗效，以期为今后对此两种疗法的选择提供参考。

**方法:**

系统地检索5个主要的医学期刊数据库：中国知网、维普网、PubMed、Embase和ISI web of science，收集时间涵盖2010年1月-2016年2月的应用电视辅助胸腔镜手术和体部立体定向放疗治疗早期NSCLC的所有文献。最后，纳入了拥有足够患者数量和放疗剂量的原创中英文研究成果。在校正了中位年龄和可手术患者人数之后，应用多变量随机效应模型比较了电视辅助胸腔镜手术和体部立体定向放疗治疗后早期NSCLC患者的总生存率和无疾病生存率。

**结果:**

纳入相同时期的14个电视辅助胸腔镜手术研究成果（包含3, 482个患者）和19个体部立体定向放疗研究成果（包含3, 997个患者）。VATS组中位年龄和随访时间分别为64岁和43.4个月，SBRT组中位年龄和随访时间分别为74岁和29.5个月。VATS组校正前1年、2年、3年、5年平均总生存率分别为93.5%、84.9%、77.0%、76.3%。SBRT组校正前1年、2年、3年、5年平均总生存率分别为89.0%、73.3%、59.0%、36.7%。VATS组校正前1年、2年、3年、5年平均无病生存率分别为93.6%、88.6%、85.6%、75.6%。SBRT组校正前1年、2年、3年、5年平均无病生存率分别为79.3%、72.1%、64.9%、58.9%。然而，以中位年龄和可手术程度进行校正之后，预期VATS组1年、2年、3年、5年总生存率为94%、92%、84%、71%，预期SBRT组1年、2年、3年、5年总生存率为98%、95%、87%、83%。预期VATS组1年、2年、3年、5年无病生存率为97%、94%、85%、75%，预期SBRT组1年、2年、3年、5年无病生存率为88%、81%、74%、63%。

**结论:**

在校正前，施以体部立体定向放疗的患者临床表现（总生存率和无病生存率）并不如施以电视辅助胸腔镜手术的患者。但考虑到年龄与可手术程度，施以体部立体定向放疗的患者年龄偏大，可手术程度偏低。以年龄和可手术程度进行校正后，我们发现两种疗法治疗早期NSCLC的总生存率和无病生存率并没有显著区别。

## Abstract (en)

**Background and objective:**

More and more chest physicians chose video-assisted thoracoscopic surgery (VATS) to treat early stage non-small cell lung cancer (NSCLC). In recent years, there is still lack of a random trial comparing the clinical outcomes of VATS and stereotactic body radiotherapy (SBRT) in treating NSCLC. To provide a reference for the choice between VATS and SBRT, in the current *meta*-analysis, we compared the clinical outcomes of these two therapies in treating NSCLC.

**Methods:**

Five major medical databases, CNKI, CPVIP (http://www.cqvip.com/), PubMed, Embase, and ISI web of science were systematically searched to identify all studies from January 2010 to February 2016 on VATS and SBRT therapies. Finally, original English or Chinese publications of stage Ⅰ and Ⅱ NSCLC with adequate patients and adequate SBRT doses were enrolled. A multivariate random effects model was used to perform a *meta*-analysis to compare overall survival and disease free survival between VATS and SBRT while adjusting for median age and operable patient numbers.

**Results:**

Fourteen VATS studies (included 3, 482 patients) and nineteen SBRT studies (included 3, 997 patients) published in the same period were eligible. The median age and follow-up duration were 64 years and 43.4 months for VATS patients and 74 years and 29.5 months for SBRT patients, respectively. The mean unadjusted overall survival rates at 1, 2, 3, and 5 years with VATS were 93.5%, 84.9%, 77.0% and 76.3% compared to 89.0% 73.3% 59.0% and 36.7% with SBRT. The mean unadjusted disease free survival rates at 1, 2, 3, and 5 years with VATS were 93.6%, 88.6%, 85.6% and 75.6% compared to 79.3%, 72.1%, 64.9% and 58.9% with SBRT. While, after adjusted for proportion of operable patients and median age, the estimate overall survival rates at 1, 2, 3, and 5 years with VATS were 94%, 92%, 84% and 71% compared to 98%, 95%, 87% and 83% with SBRT. And the estimate disease free survival rates at 1, 2, 3, and 5 years with VATS were 97%, 94%, 85% and 75% compared to 88%, 81%, 74% and 63% with SBRT.

**Conclusion:**

Before adjustment, the SBRT group showed worse clinical outcomes (overall survival and disease free survival) than VATS group. When take consider of median age and operability, the patients with SBRT differ substantially from patients treated with VATS. After adjustment of median age and operability, there are no significant differences between these two therapy in treating NSCLC.

肺癌是导致肿瘤患者死亡的最常见因素^[1]^。早期非小细胞肺癌（non-small cell lung cancer, NSCLC）的推荐疗法为肺叶切除术，但是很多Ⅰ期NSCLC患者由于并发症或拒绝手术导致其无法进行肺叶切除术^[2]^。

微创叶切除手术已经成为胸外科手术的先进技术。与传统开胸手术相比，电视辅助胸腔镜手术（video-assisted thoracoscopic surgery, VATS）可以避免较大的损伤，属于微创手术范畴。电视辅助胸腔镜肺叶切除手术对早期NSCLC患者来说是可接受的^[3, 4]^。

体部立体定向放疗（stereotactic body radiotherapy, SBRT）是早期NSCLC患者的另一选择。这类患者往往由于身体条件限制或心理抵触而不能进行手术。非随机试验研究已经表明体部立体定向放疗的局部控制和总生存率已经可以和叶切除术相当^[5-7]^。另外，体部立体定向放疗治疗周围肺转移患者时可以实现较高的局部控制率和较低毒性反应^[8-10]^。

到目前为止，仍然缺乏随机对照试验来比较电视辅助胸腔镜肺叶切除手术和体部立体定向放疗的临床疗效。倾向性分析使得从众多基线因素中匹配两个相似组进行比较成为可能。电视辅助胸腔镜肺叶切除手术和体部立体定向放疗已经在世界范围内广为应用，由此，我们使用混合效应模型比较了早期NSCLC患者经以上两种疗法治疗后的总生存率和无病生存率。

## 1 材料和方法

### 1.1 文献检索策略

我们检索了中国知网、维普网、PubMed、Embase和ISI web of science等多种电子期刊数据库。为了方便比较，电视辅助胸腔镜手术和体部立体定向放疗研究均来自这些数据库，并且发表年基本相同。为了尽可能有效地识别相关文献，文献检索遵循图 1所示的标准流程，此流程展示了每步所得结果。

**1:**
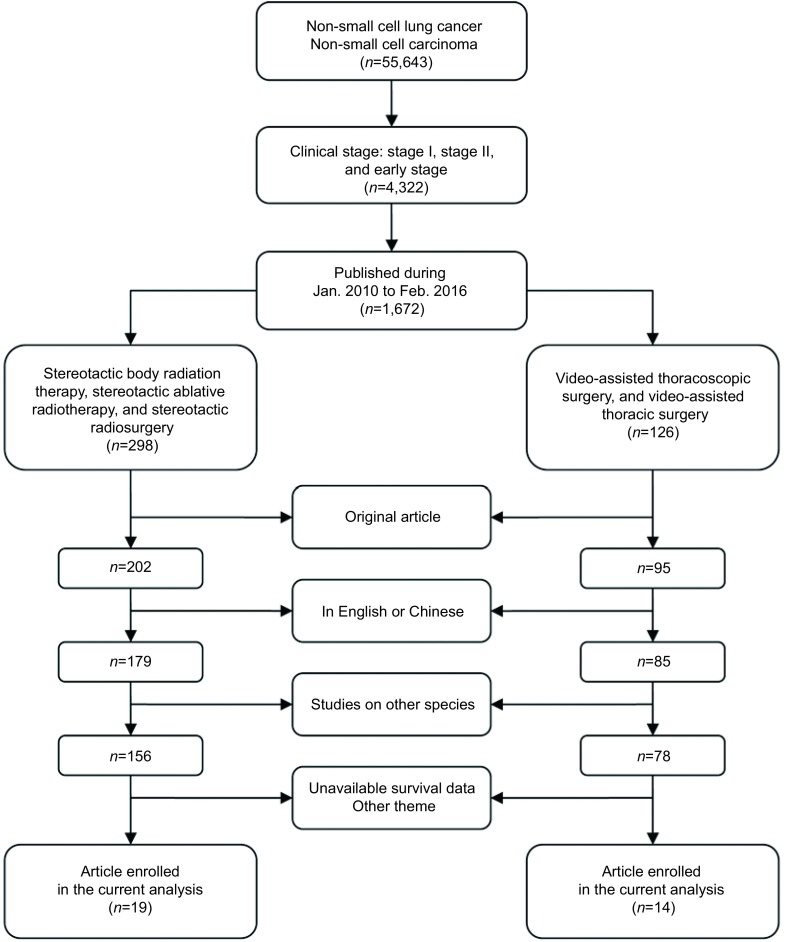
文献筛选流程图 Election strategy of studies enrolled in the current *meta*-analysis

### 1.2 文献纳入与排除标准

纳入与排除严格遵循如下标准：①2010年1月-2016年2月间以中文或英文发表的原始文献；②早期NSCLC限制在临床Ⅰ期、Ⅱ期的NSCLC，分期标准应遵循美国癌症联合委员会颁布的癌症分期标准第7版；③电视辅助胸腔镜手术缩写为VATS（video-assisted thoracoscopic surgery），与全胸腔镜手术等同。早期NSCLC手术过程可包括全切除术，如叶切除、二叶切除术，全肺或限制肺切除术，如次肺叶切除术、肺节切除术、楔状切除术。④体部立体定向放疗缩写为SBRT，与定向消融治疗和立体定位放射手术等同。根据体部立体定向放疗的定义，以低分割放射治疗剂量＞8 Gy、次数≤8才可算作体部立体定向放疗，不符合要求的研究均不予采用^[11, 12]^。⑤报道了总生存率和/或无病生存率数据或该数据可根据发表结果获得的研究。某些作者有多篇研究报道符合文献纳入标准的，为了减少数据的重复，每篇文献均进行评估，选取其中材料最新、患者数最大，生存率数据最全的研究^[13, 14]^。

### 1.3 数据提取

我们独立对所有文献进行数据提取。从体部立体定向放疗研究中提取的数据应包括第一作者、发表年、研究类型、研究年限、患者总数、可手术患者百分比、男性百分比、中位年龄、临床分期、病理结果、肿瘤大小、剂量、BED_10_、随访时间、总生存率和无进展生存率。从电视辅助胸腔镜手术研究中提取的数据应包括第一作者、发表年、研究类型、研究年限、患者总数、男性百分比、中位年龄、手术过程、临床分期、随访时间、总生存率和无病生存率。不一致数据由两位评审协商一致后确定。研究类型主要为回顾性研究（14篇电视辅助胸腔镜手术研究中有12篇回顾性研究，19篇体部立体定向放疗研究中有18篇回顾性研究）。生存率数据如果在文献中没有直接说明，则使用Engauge Digitizer V4.1（Slashdot Media）从生存曲线中读取。同时，提取的生存率数据与文献中已有数据进行比较，确保最大误差为±0.05。

### 1.4 数据统计和分析

*Meta*分析的主要目的是比较施以SBRT疗法的早期NSCLC患者与施以VATS患者的总生存期。作为第一步，为衡量每个结果所有的统计数据都进行了计算处理。这些统计数据并不是比较结果（由于患者人群差异很大），而是要总结患者群体治疗后的结果。多元随机效应模型可以用来对针对潜在的混杂因素校正后的生存数据进行*meta*分析^[15, 16]^。预测校正后的生存率，先进行ln-minus-ln转换，这里的ln（time）是作为SBRT或者VATS疗法年龄和可手术程度固定共变量的协变量。预测的生存曲线结果符合韦伯族。研究特异的时间斜率和截距的随机效应用来联系各个研究。本*meta*分析应用了非结构化研究间协方差矩阵。参数以迭代方式确定，重复调用SAS V9.4（Cary, NC）里的混合式模型方差分析以更新研究内相关关系。使用这个混合随机效应模型，我们计算了以年龄和可手术患者百分比校正后的两种疗法的风险比，同时预测了平均年龄为70、可手术患者百分比为100%时的生存率。由于文献中通常不会提供生存数据的标准差和置信区间，我们通过已经给出的生存率和相应的随访人数计算了研究内方差和协方差矩阵^[16, 17]^。随访人数根据中位随访时间和一个假定随访时间指数耗损模型来确定。对于没有报道中位随访时间的研究，我们以其他研究报道的随访时间的中位数来确定。置信区间基于研究水平表现的变异性来计算，反映了研究间异质性和抽样变异。

## 2 结果

### 2.1 文献检索及情况

共有67篇VATS研究和121篇SBRT研究符合最初的筛选标准，下载获取到全文，进行进一步全文筛选。其中23篇前瞻性研究和165篇回顾性研究，总计患者人数为120, 930。排除重复的研究之后，没有报道生存数据、研究其他物种的研究均被排除。最终本篇*meta*分析纳入14篇VATS研究（其中2篇为前瞻性研究）包含3, 482例患者（表 1）和19篇SBRT研究（其中1篇为前瞻性研究）包含3, 997例患者（表 2）。总体来说，接受VATS治疗的患者明显比接受SBRT治疗的患者年轻[分别为（64.0±5.4）岁和（71.4±6.7）岁，*P*＜0.005]。而两组男性比例差异不明显（分别为57.3%和59.9%，*P*=0.255）。VATS治疗组随访时间较SBRT组长（分别为43.4个月和28.5个月）。

**1:** 本篇*meta*分析纳入的2010年1月-2016年2月14个VATS研究 14 studies on VATS from January 2010 to February 2016 included in the current *meta*-analysis

Author	Pub. year	Res. type	Research year range	Total pts	Male (%)	Med.age	Sur. Pro. (ptsn)	Clinical stage				F/U (mo)
								Ⅰa	Ⅰb	Ⅱa	Ⅱb	
Kim, *et al*^[18]^	2010	R	2003-2008	436	/	/	LR (436)	248	188	13	44	> 20
Puri, *et al*^[19]^	2010	R	2000-2006	841	/	65	/	621		220		> 24
Sugi, e*et al*^[20]^	2010	R	2001-2004	139	36.2	64	LLR (43), LR (95)	128		11		> 60
Gao, *et al*^[21]^	2011	R	2006-2009	89	82	61	LR (89)	70		19		2-48
Yamashita, *et al*^[22]^	2011	R	2003-2008	109	60.6	70	LLR (38), LR (71)	83	26	/	/	> 27.5
Zhao, *et al*^[23]^	2012	P	2010-2011	46	80.4	60	LR (46)	39		7		10.2
Zhao, *et al*^[24]^	2012	P	2009-2010	36	63.9	/	/	27		9		> 36
Jiang, *et al*^[25]^	2012	R	2005-2008	160	51.9	61	LR (160)	46	83	3	10	> 60
Marty-Ané, *et al*^[26]^	2013	R	1996-2011	312	65.1	62	LR (364)	183	90	10	29	> 60
Nakano, *et al*^[27]^	2014	R	2010-2012	464	55.7	68	LR (464)	/	/	/	/	/
Murakawa, *et al*^[28]^	2015	R	2001-2010	101	51.5	69	LR (101)	51	30	18	2	60
Nwogu, *et al*^[29]^	2015	R	2004-2010	175	48	69	LR (175)	/	/	/	/	60
Zhou, *et al*^[30]^	2015	R	2006-2012	550	38	68	LR (493), LLR (57)	/	/	/	/	> 32.4
Zheng, *et al*^[31]^	2015	R	2009-2010	24	54.2	51	LR(24)	/	/	/	/	1-36
Pub.year: publication year; Res.type: research type (P: prospective; R: retrospective); Research yrr: research year range, the year range of patients who is enrolled in the study; Total pts: total patients; Med.age: median age; Sur.Pro.(ptsn): surgery procedure (patients number); F/U (mo): follow-up period (months); /: not reported or obscure; VATS: video-assisted thoracic surgery.												

**2:** 本篇*meta*分析纳入的2010年1月至2016年2月19个SBRT研究 19 studies on SBRT from January 2010 to February 2016 included in the current *meta*-analysis

Author	Res. type	Research year range	Total pts	Opp (%)	Male (%)	Med. age	Clinical stage				Path. (%)	Size (mm)	Dose range	BED10	F/U(mo)
							Ⅰa	Ⅰb	Ⅱa	Ⅱb					
Baba, *et al*^[32]^	R	2004-2008	124	32.4	67.7	77	87	37	/	/	91.9	m27	44, 48, 52	92.4, 105.6, 119.6	26(7-66)
Zhang, *et al*^[33]^	P	2006-2008	30	0	60	62	2	5	23		100	/	40-50	57.6-100	> 24
Matsuo, *et al*^[34]^	R	1998-2007	101	36.6	73.3	77	33	40	28	/	100	≤40	48	105.6	31.4
Chang, *et al*^[35]^	R	2005-2009	130	26.2	51.5	74	112	18	/	/	/	< 50	50	112.5	26
Grills, *et al*^[10]^	R	1998-2010	483	13	52	74	304	159	10	5^*^	64	/	20-64	132	19.6(1.2-87.6)
Zheng, *et al*^[36]^	R	2004-2008	54	/	64.8	56	/	/	/	/	100	/	50, 56	100, 134.4	> 36
Du, *et al*^[37]^	R	2007-2010	52	/	69.2	62	/	/	/	/	100	/	20-36	30-57.6	> 24
Senthi, *et al*^[38]^	R	2003-2011	676	31	61	73	/	/	/	/	/	/	54-60	105-180	32.9(14.9-50.9)
Shibamoto, *et al*^[39]^	R	2004-2008	180	33.3	68.3	77	128	52	/	/	100	12-50	44, 48, 52	92.4, 105.6, 119.6	36
Takeda, *et al*^[40]^	R	2005-2011	115	27	78	78	/	/	/	/	100	/	40-50	72-100	21.2(6-63.7)
Ren, *et al*^[41]^	R	2008-2011	20	25	75	76	7	3	2	8	100	< 30, 9; ≥30, 11	48	51-83	> 36
Badiyan, *et al*^[42]^	R	2004-2009	120	0	52	74	/	/	/	/	80.8	/	54	151.2	29
Zhang, *et al*^[43]^	R	2006-2009	52	36.5	67.3	66	22	11	13	6	100	/	48-56	64.8-154.8	> 36
Ricardi, *et al*^[44]^	R	2003-2011	196	7.7	74.5	75	155	41	/	/	/	24.8 (9-50)	45-60	100-151.2	30
Davis, *et al*^[45]^	R	2004-2014	111	15.3	53.1	69	/	/	/	/	/	22.2	37.5, 48	93.6, 105.6	17(1-72)
Davis, *et al*^[46]^	R	2004-2013	723	48	48%	76	/	/	/	/	/	/	54 (10-80)	151.2 (20-240)	12(1-87)
Kohutek, *et al*^[47]^	R	2006-2012	211	/	43.6	77	/	/	/	/	100	/	> 45	< 100 (19.2%), ≥100 (80.8%)	25.2(4.3-75.2)
Schanne, *et al*^[48]^	R	2003-2011	567	/	70.7	72	297$	223	30		/	/	37.5 (12-64)	72 (43-180)	18.8
Wu, *et al*^[13]^	R	2004-2010	52	/	55.8	61	/	/	/	/	100	/	40-50	80-100	> 36
Opp: operability percentage of patients with operable diseases; Path (%): percentage of disease with pathological confirmation; BED_10_: biological equivalent dose with *α*/*β*=10, calculated based on the linear-quadratic equation BED_10_=nd [1+d/(*α*/*β*)], where n and d represent the number of fractions and the dose per fraction, respectively.															

### 2.2 校正前总生存率和无病生存率

1年、2年、3年、5年总生存率是所有研究中最普遍的终点指标。SBRT组校正前1年、2年、3年、5年平均总生存率分别为89.0%、73.3%、59.0%、36.7%。VATS组校正前1年、2年、3年、5年平均总生存率较SBRT组高，分别为93.5%、84.9%、77.0%、76.3%。VATS组校正前1年、2年、3年、5年平均无病生存率分别为93.6%、88.6%、85.6%、75.6%。SBRT组校正前1年、2年、3年、5年平均无病生存率分别为79.3%、72.1%、64.9%、58.9%（图 2）。不考虑患者的诸如年龄差异，VATS组的总生存率和无病生存率都比SBRT组高（图 3）。

**2:**
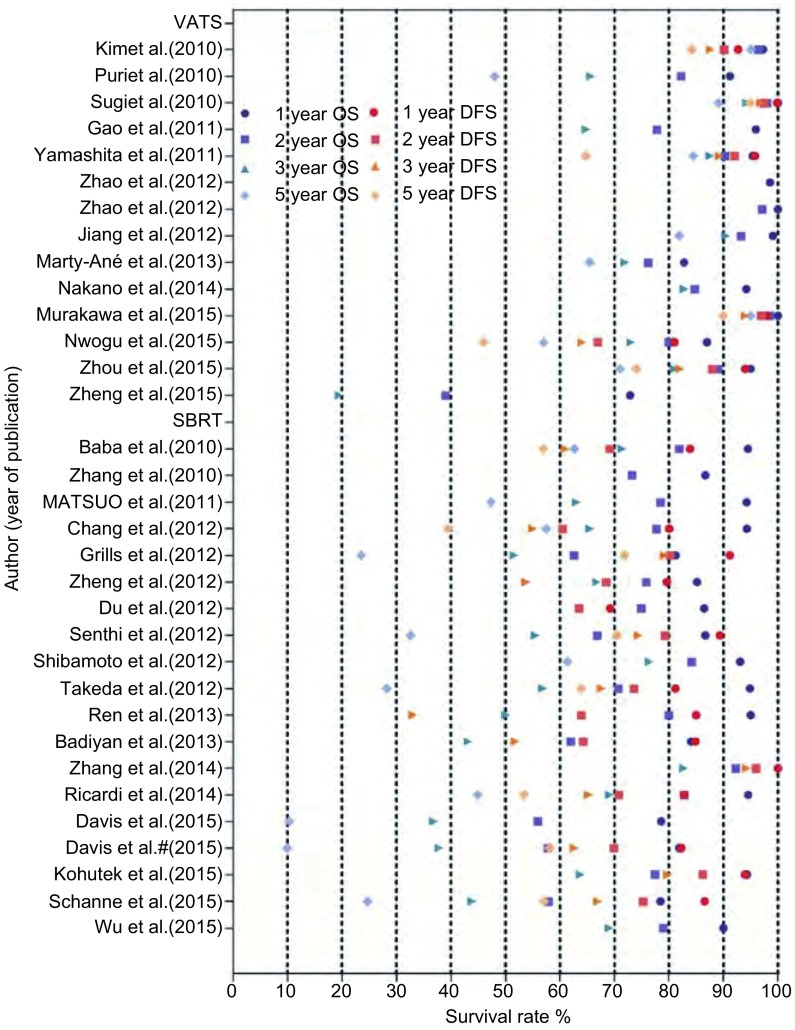
VATS组和SBRT组早期非小细胞肺癌患者的总生存率和无病生存率比较。该图展示了被纳入到此*meta*分析中所有研究的1年、2年、3年、5年总生存率和无病生存率。 Overall survival (OS) and disease free survival (DFS) comparison between VATS and stereotactic body radiation therapy (SBRT) group for early-stage non-small cell lung cancer (NSCLC). The figure shows a pooled presentation of 1-, 2-, 3-and 5-year OS and DFS from all studies enrolled in this *meta*-analysis with corresponding data available.

**3:**
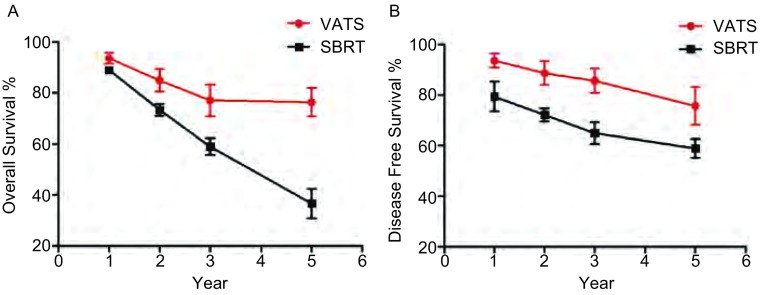
VATS组和SBRT组早期（Ⅰa、Ⅰb、Ⅱa、Ⅱb）NSCLC患者总生存率（A）和无病生存率（B）比较 OS (A) and DFS (B) comparison between VATS and SBRT group in early-stage (stage Ⅰa, Ⅰb, Ⅱa, and Ⅱb) NSCLC

### 2.3 以年龄和可手术患者比例校正后的总生存率和无病生存率

除2篇研究^[18, 24]^之外，本*meta*分析纳入的所有研究均给出了中位年龄。VATS组中位年龄为64岁，SBRT组中位年龄为74岁。这说明两组疗法患者的年龄或许是其中的混杂因素。考虑到年龄的影响，总生存率与报道的患者中位年龄相关（*P*＜0.05，图 4A、图 4B、图 4C）。对于无病生存率可以得到相似的结果——无病生存率与中位年龄呈负相关（*P*＜0.05，图 4D、图 4E、图 4F）。

**4:**
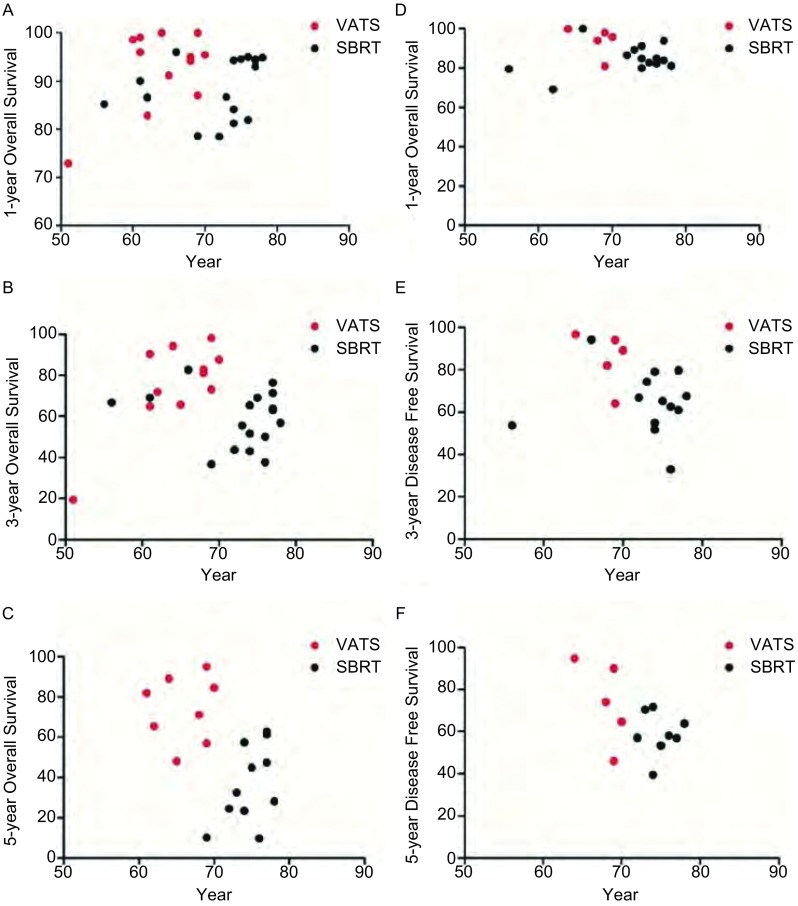
VATS组和SBRT组年龄依赖的总生存率和无病生存率的比较。预测的VATS组和SBRT组1年总生存率（A）、3年总生存率（B）、5年总生存率（C）、1年无病生存率（D）、3年无病生存率（E）、5年无病生存率（F）。该结果以中位年龄为基础。 Age-dependent OS and DFS comparison in VATS and SBRT group. Estimated 1-year OS (A), 3-year OS (B), 5-year OS (C), 1-year DFS (D), 3-DFS (E), 5-DFS (F) for VATS versus SBRT by median trial age.

14篇SBRT研究（3, 061例患者）中报道了接受SBRT治疗的患者的可手术比例，平均为23.7%（0-48%，中位数为14.2%）。平均总生存率随着可手术比例的增高而增大（*P*＜0.05，图 5A、图 5B），可手术比例与3年、5年总生存率相应的*Spearman*相关系数为0.66和0.58。然而无病生存率并不存在明显的相关性（*P*＞0.05，图 5C、图 5D）。

**5:**
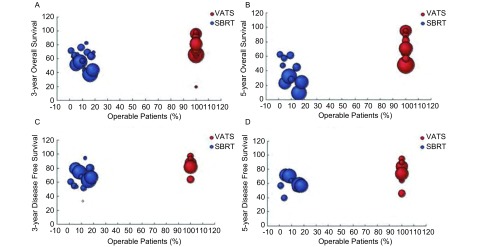
VATS组和SBRT组可手术性依赖的总生存率和无病生存率的比较。预测的VATS组和SBRT组3年总生存率（A）、5年总生存率（B）、3年无病生存率（C）、5年无病生存率（D）。球体大小表示相应研究中患者数量。该结果以中位年龄为基础。 Operability-dependent OS and DFS comparison in VATS and SBRT group. Estimated 3-year OS (A), 5-year OS (B), 3-year DFS (C), 5-year DFS (D) for VATS versus SBRT by median trial age by proportion. Dot sizes are proportional to the number of patients in specific studies.

由于这些数据的非随机化特性，因此在进行研究间比较时必须对其存在的混杂因素进行控制或校正。为此，我们必须使用文献中已报道的数据。一个普遍存在的混杂因素便是并发症，即影响患者可否进行手术的因素，也将影响最终的生存率。此外，我们还将年龄作为其中一个混杂因素，因为他——①被普遍报道；②在VATS组和SBRT组存在统计学差异；③与生存率直接相关！当我们用一个回归模型对这两个混杂因素进行校正之后，我们发现两组的生存率不再存在明显差异（HR=1.89，95%CI：0.38-9.51，*P*=0.44，表 3）。同时，无病生存率也不存在明显差异（HR=0.58，95%CI：0.12-2.72，*P*=0.49，表 3）。由拟合的回归模型，我们计算了中位年龄为70岁、可手术比例为100%时的1年、2年、3年、5年预期总生存率（表 4）。SBRT组比VATS组有较高的总生存率，但其无病生存率较VATS组低。然而，这些结果并无统计学意义。

**3:** 多元混合效应模型中VATS组和SBRT组总生存率和无病生存率的风险比 Hazard ratio (VATS to SBRT) of overall survival and disease free survival estimates from the multivariate mixed effects model

Model	Hazard ratio	95%CI	*P*
Overall survival			
Treatment			0.39
VATS to SBRT	1.89	0.38-9.51	0.44
Median age	1.22	1.10-1.36	0.000, 3
Percentage of operability	0.89	0.81-0.93	0.02
Disease-free survival			
Treatment			0.49
VATS to SBRT	0.58	0.12-2.72	0.49
Median age	0.96	0.82-1.12	0.61
Percentage of operability	1.12	0.89-1.41	0.33

**4:** 基于模型（中位年龄为70岁，可手术比例为100%）的总生存率和无病生存率预测 Model-based overall survival and disease free survival estimates for a trial with median age 70 and 100%

Model	Year	SBRT			VATS	
		Estimate	95%CI		Estimate	95%CI
Overall survival	1	0.98	0.97-0.99		0.94	0.89-0.99
	2	0.95	0.90-1.00		0.92	0.85-0.99
	3	0.87	0.77-0.97		0.84	0.83-0.85
	5	0.83	0.71-0.95		0.71	0.63-0.79
Disease free survival	1	0.88	0.83-0.93		0.97	0.94-1.00
	2	0.81	0.74-0.88		0.94	0.88-1.00
	3	0.74	0.63-0.85		0.85	0.79-0.91
	5	0.63	0.56-0.70		0.75	0.61-0.89

## 3 讨论

校正前，接受SBRT治疗的患者与接受VATS治疗的患者相比有更严重的临床结果，包括总生存率（overall survival, OS）和无病生存率（disease-free survival, DFS）。然而，接受SBRT治疗的患者和接受VATS治疗的患者在平均年龄和可手术比例上有大不同。在校正了以上两个混杂因素以后，接受SBRT治疗的患者比接受VATS治疗的患者有更高的总生存率。同时，接受VATS治疗的患者比接受SBRT治疗的患者有更高的无病生存率。他们之间相比并没有统计学差异。

SBRT疗法和VATS疗法的混杂因素复杂，包括临床分期、肿瘤大小、放疗剂量，支气管肺泡癌，病理确证，NSCLC外围或中央定位，可手术程度以及患者的年龄。Baba等和Ricardi等^[32, 49]^的研究表明尽管肿瘤大小的差异，Ia期和Ib期肿瘤之间的局部控制率没有区别。然而，对于稍大的肿瘤增加SBRT剂量是否有益有待于进一步研究。与其他NSCLC亚型相比，细支气管肺泡癌（bronchioloalveolarcarcinoma, BAC）在SBRT治疗后似乎有相似的失败模式和生存模式，不过BAC可能会增加远处转移的风险^[42]^。病理确诊的NSCLC患者和那些暂定为肺癌（被认为是最有可能的NSCLC）之间在总体存活率上没有统计学差异^[50]^。SBRT治疗的中央肺肿瘤患者的总生存率是可观的^[45]^。此外，中心位置的NSCLC与外围的NSCLC相比有更好的总生存率和局部无进展生存率。中心位置的NSCLC与周边位置的NSCLC的疗效是相近的^[48]^。

在本篇*meta*分析中，我们发现年龄和可手术比例的确影响VATS和SBRT治疗早期NSCLC患者时的总生存率和无病生存率。

庞景灼等^[51]^在他们的研究中比较了电视辅助胸腔镜肺段切除和肺叶切除手术治疗早期NSCLC患者的短期疗效，发现术后1年内，患者未出现死亡病例，未发现肿瘤复发转移情况，肺段切除术组在术后1年肺功能减少率明显优于肺叶切除组。他们指出，肺段切除术手术时间较肺叶切除术长，但其在各方面效果优于或等于肺叶切除术。杜恒等^[52]^对胸腔镜手术在中国地市级医院胸外科应用现状进行问卷调查，得出结论：①对胸腔镜手术的看法：89.62%（164/183）的医师认为胸腔镜手术优点主要是创伤小且康复快，缺点是费用高（76.50%, 140/183）；71.04%（130/183）的医师认为肺癌患者行胸腔镜肺叶切除术后生活质量高，而仅有12.57%（23/183）认为5年生存率优于开胸手术；60.11%（110/183）的医师认为国内普及水平不如美国，但个别中心的水平优于或等同于美国；52.46%（96/183）认为胸腔镜手术可应用于局部晚期肺癌的治疗。②胸腔镜肺叶切除术学习情况：学习班或短期培训（32.24%, 59/183）是胸腔镜肺叶切除术的最佳学习途径，开胸-小切口-腔镜（60.66%, 111/183）是主要学习过程，而单向式（54.64%, 100/183）是主要技术，其学习曲线是至少30例（26.78%, 49/183）；③胸腔镜应用情况：被调查医师所在的183家医院均开展胸腔镜手术，胸部良性疾病是最初开展（81.42%, 149/183）的主要病种，肺癌肺叶切除术最初开展的困难是手术室及团队配合差（39.34%, 72/183）和医师技术未成熟（36.07%, 66/183）；且胸腔镜技术的进一步优化（118/183, 64.48%）是其发展方向。因此，我国VATS和SBRT的进一步发展将十分有助于提高我国早期NSCLC患者生存率。

考虑到年龄和可手术比例，VATS和SBRT疗法显示出微弱的差别。两种疗法对于治疗早期NSCLC是可行且有效的。然而，仍需要随机前瞻性试验来比较两者的临床疗效。
